# The Role of Specific Chemokines in the Amelioration of Colitis by Appendicitis and Appendectomy

**DOI:** 10.3390/biom8030059

**Published:** 2018-07-20

**Authors:** Rajkumar Cheluvappa, Dennis G. Thomas, Selwyn Selvendran

**Affiliations:** 1 Department of Medicine, St. George Clinical School, University of New South Wales, Sydney, NSW 2052, Australia; 2 Biological Sciences Division, Pacific Northwest National Laboratory, Richland, WA 99352, USA; dennis.thomas@pnnl.gov; 3 Department of Surgery, St. George Hospital, Kogarah, NSW 2217, Australia; tselvendran@hotmail.com

**Keywords:** appendectomy, appendicitis, chemokine, chemokine receptor, CCL5, CCL7, CCL8, CCL17, CCL20, CCR10, CXCL11, inflammatory bowel disease, ulcerative colitis

## Abstract

The appendix contains abundant lymphoid tissue and is constantly exposed to gut flora. When completed at a young age, appendicitis followed by appendectomy (AA) prevents or significantly ameliorates Inflammatory Bowel Diseases (IBDs) in later life. Inflammatory bowel disease comprises Crohn’s disease and ulcerative colitis. Our murine AA model is the only existing experimental model of AA. In our unique model, AA performed in the most proximal colon limits colitis pathology in the most distal colon by curbing T-helper 17 cell activity, diminishing autophagy, modulating interferon activity-associated molecules, and suppressing endothelin vaso-activity-mediated immunopathology. In the research presented in this paper, we have examined the role of chemokines in colitis pathology with our murine AA model. Chemokines are a family of small cytokines with four conserved cysteine residues. Chemokines induce chemotaxis in adjacent cells with corresponding receptors. All 40 known chemokine genes and 24 chemokine receptor genes were examined for gene expression levels in distal colons three days post-AA and 28 days post-AA. At 28 days post-AA, the chemokine gene *CCL5* was significantly upregulated. Furthermore, Gene Set Enrichment Analysis (GSEA) showed upregulation of seven *CCL5*-associated gene-sets 28 days post-AA in contrast to just one gene-set downregulated at the same time-point. The chemokine gene *CXCL11* was significantly upregulated three days post-AA and 28 days post-AA. Evaluation using GSEA showed upregulation of six *CXCL11*-associated gene sets but no downregulation of any gene set. At 28 days post-AA, *CCL17* gene expression was significantly downregulated. There was no expression of any chemokine receptor gene three days post-AA, but *CCR10* was the only chemokine receptor gene that displayed differential gene expression (upregulation) 28 days post-AA. No *CCR10*-associated gene set was upregulated in GSEA in contrast to one downregulated gene set. Our analysis resulted in identifying three new therapeutic targets towards ameliorating colitis: CCL5, CXCL11, and CCL17. While CCL5 and CXCL11 are good therapeutic chemokine candidates to be exogenously administered, CCL17 is a good candidate chemokine to competitively inhibit or limit colitis pathology.

## 1. Introduction

Appendicitis is the most common abdominal emergency requiring surgery [1]. The appendix has abundant lymphoid tissue and is constantly exposed to intestinal pathogens and commensals. However, the complex interplay between genetic proclivity, gut flora, and intestinal immunity in Inflammatory Bowel Diseases (IBD) comprising ulcerative colitis and Crohn’s disease is not clear. The critical role of appendicitis followed by appendectomy (AA) in ameliorating or preventing the development of human ulcerative colitis [2,3,4] and Crohn’s disease [3,5] is limited to patients having surgery before turning 20 years of age [4]. Using our murine AA model [6], we have previously demonstrated that AA performed in the most proximal colon limits colitis pathology by inducing the activities below in the most distal colon.

1. Substantial curbing of T-helper 17 cell-recruitment, -differentiation, -activation, and -effector interleukin expression [7]. Th17 cell activity and migration to the site of colitis is also suppressed by AA through the downregulation of pro-inflammatory Th17 cell recruitment-factor CCL20 [8].
2. Global suppression of autophagy gene expression and gene-set expression [9].
3. Late suppression of endothelin-related genes and gene-sets, specifically endothelins (ET-1 and ET-2), and endothelin converting enzyme B [10].
4. Upregulation or downregulation of genes and gene-sets specific to interferon activity [11].

In this work, we have examined the role of chemokines in colitis pathology with our murine AA model. Chemokines are a family of small CHEMOtactic cytoKINES (CHEMOKINES), which induce chemotaxis in adjacent cells with corresponding receptors [12]. The balance of chemokines is crucial for immunological homeostasis [13] and derangements in their activity have significant roles in immunopathology [14], which makes them good potential targets in diseases with inflammatory and immunological pathophysiology like ulcerative colitis [15,16]. Chemokines are small (8–10 kDa), have 4 conserved cysteine residues participating in the formation of their 3-dimensional morphology, and are classified into 4 subfamilies, which include CXC, CC, CX3C, and XC [12]. Chemokines act through the interaction with G protein-linked transmembrane receptors on target cell membranes [12].

Elaboration of information on all known chemokines and chemokine receptors are redundant, irrelevant, and beyond the scope of this paper. In this paper, we have focused on briefly describing the known roles of chemokines and chemokine receptor(s), which showed statistically significant expression changes. The chemokine CCL5 is a chemoattractant for monocytes, memory T-helper cells, and eosinophils [17]. It is also an eosinophil-activator and induces histamine release from basophils [17]. The chemokine CCL7 is a chemoattractant for macrophages and a substrate of extracellular matrix metalloproteinase 2 [18]. The chemokine CCL8 is a chemoattractant for monocytes, lymphocytes, basophils, and eosinophils [19]. The chemokine CCL17 is a chemo-attractant for T lymphocytes, which assisted in their activation [20]. The chemokine CCL20 is a chemo-attractant for lymphocytes and neutrophils, which may assist mucosal lymphoid tissues to attract lymphocytes and dendritic cells towards epithelial cells [8]. The chemokine CXCL11 is a chemo-attractant for interleukin-activated T cells, which are induced by gamma and beta interferons [21]. The chemokine receptor CCR10 is the receptor for the chemokine CCL27, which is a chemo-attractant for memory T lymphocytes [19].

Gene expression analysis strategies may highlight differences in individual gene expression between two experimental groups, but do not deal with the complex reality of cellular processes cohesively effecting changes. These concerted cumulative changes, however, become evident as differential expression of groups of genes (gene-sets). While gene changes may be minimal at the level of individual genes, they can be substantial at the level of gene-sets. To this end, we have used Affymetrix^®^ microarray analysis, Reverse Transcription-Polymerase Chain Rection (RT-PCR), and gene set enrichment analysis (GSEA) [22] to characterize the roles of individual chemokines, chemokine receptors, and associated pathophysiological pathways involved in the protective anti-colitic effect of AA in our model.

## 2. Results

### 2.1. Individual Distal Colonic Gene Expression of 40 Chemokine Genes Three Days Post-AA and 28 Days Post-AA

Microarray expression levels of 40 chemokine genes [13] from distal colons three days post-AA and 28 days post-AA were examined (Table 1). At three days post-AA, *CXCL11* was significantly upregulated and *CCL20* was significantly downregulated (* *p* value < 0.05). At 28 days post-AA, *CCL5* and *CXCL11* were significantly upregulated and *CCL17* and *CCL20* were significantly downregulated (* *p* value < 0.05).

### 2.2. Individual Distal Colonic Gene Expression of 24 Described Chemokine Receptor Genes Three Days Post-AA and 28 Days Post-AA

Microarray gene expression levels of 24 chemokine receptor genes [23] from distal colons three days post-AA and 28 days post-AA were examined (Table 2). There was no gene expression of any chemokine receptor gene three days post-AA. However, there was gene expression of most chemokine receptor genes 28 days post-AA. The chemokine receptor gene *CCR10* was the only chemokine receptor gene that displayed differential gene expression 28 days post-AA specifically upregulation (* *p* value < 0.05).

### 2.3. Differentially Regulated Distal Colonic Gene Sets Associated with Differentially Regulated Individual Chemokine and Chemokine Receptor Genes 28 Days Post-AA

The Gene Set Enrichment Analysis with stringent criteria (False Discovery Rate or FDR < 5% and *p* Value < 0.001) revealed that 7 *CCL5*-associated gene-sets were upregulated 28 days post-AA in contrast to one downregulated gene set (Table 3). No *CCL17*-associated gene sets were upregulated or downregulated. One *CCL20*-associated gene set was upregulated in contrast to no gene sets downregulated. Six *CXCL11*-associated gene sets were upregulated in contrast to no gene sets downregulated. No *CCR10*-associated gene sets were upregulated in contrast to one gene set downregulated.

### 2.4. Differentially Regulated 28 Days Post-AA Gene-Sets Associated with Chemokines CCL7 and CCL8 Which Showed High-Fold-Change Gene Expression in the Distal Colon Three Days Post-AA

Gene Set Enrichment Analysis was also done on *CCL7* and *CCL8* 28 days post-AA, which is due to the fact that the individual genes *CCL7* and *CCL8* showed high fold-changes in AA without statistical significance (Table 4). Using stringent criteria (FDR < 5% and *p* value < 0.001), it was found that no *CCL7*-associated gene sets were upregulated 28 days post-AA in contrast to one gene set downregulated. Two *CCL8*-associated gene sets were upregulated 28 days post-AA in contrast to no gene sets downregulated.

### 2.5. RT-PCR Expression Study of CCL7 and CCL8 Chemokines Three Days Post-AA and 28 Days Post-AA

**Figure 1:**
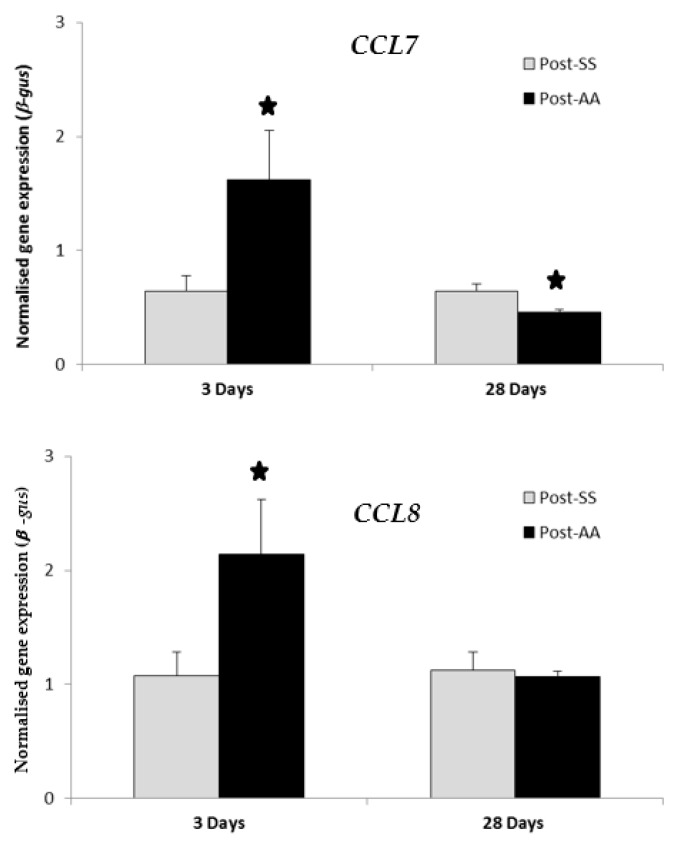
RT-PCR expression study of CCL7 and CCL8 chemokines three days post-AA and 28 days post-AA. RT-PCR transcript levels of chemokines *CCL7* and *CCL8* were compared in AA mice versus SS mice at either three days or 28 days after the second surgery. Expression of both *CCL7* and *CCL8* were significantly increased by AA three days after the second surgery when compared to controls (SS). Expression of *CCL7* was significantly decreased by AA 28 days after the second surgery when compared to controls (SS). Expression of *CCL8* was decreased (not statistically significant) by AA 28 days after the second surgery when compared to controls (SS). AA, Appendicitis-appendectomy, SS, Sham-sham. * *p* value < 0.05.

RT-PCR transcript levels of chemokines *CCL7* and *CCL8* were compared in SS mice versus AA mice at either three days or 28 days after the second surgery (Figure 1). Expression of both *CCL7* and *CCL8* were significantly increased by AA (*p* value < 0.05) three days after the second surgery when compared to SS controls. Expression of *CCL7* was significantly decreased by AA (*p* value < 0.05) 28 days after the second surgery when compared to SS controls. Expression of *CCL8* was decreased (not statistically significant) by AA 28 days after the second surgery when compared to SS controls. 

## 3. Discussion

Using a novel murine appendicitis model we developed [6], we had shown that AA provided significant protection against subsequent experimental colitis by curtailing T-helper 17 cell-recruitment, cell-differentiation, cell-activation, and cell-effector interleukin expression [7] by suppressing autophagy gene expression and gene-set expression [9], by suppressing endothelin-related genes and gene-sets [10], and by upregulating or downregulating genes and gene-sets specific to IFN activity [11].

Our microarray data is robust due to the experimental design, which is evidentially buttressed by the validation process. Our Affymetrix microarray experiments involved test samples from four individual mice for the three-day post-SS time-point and four individual mice for the for the three-day post-AA time-point. Our Affymetrix microarray experiments involved test samples from three individual mice for the 28-day post-SS time-point and three individual mice for the 28-day post-AA time-point. None of the samples were pooled. RNA from each mouse colon was taken individually through the microarray process and our microarray data displays differential expression only if the colonic RNA from each mouse from an experimental group showed differential expression compared to the colonic RNA from each mouse from the control group [24].

Our microarray data was validated by two additional methodologies. First, our study was validated by quantitative RT-PCR of 14 selected genes from various groups [24]. Additionally, we also conducted quantitative RT-PCR time-course experiments of these genes at three different time-points [24]. Second, the enrichment of gene-sets via GSEA were shown to corroborate differential expression of individual genes using stringent criteria (FDR < 5% and *p* value < 0.001). Our GSEA data was robust because gene-set expression from the colons of each mouse were individually computed. The GSEA data represents common gene sets that were differentially expressed in the colonic RNA from each mouse in an experimental group in comparison to each mouse in the control group (FDR < 5% and *p* value < 0.001) [24].

This work examined all known chemokine genes [13] for gene expression levels in distal colons three days post-AA and 28 days post-AA. The chemokines and chemokine receptor(s) that showed statistically significant expression changes in our study were CCL5, CCL7, CCL8, CCL17, CCL20, CXCL11, and CCR10.

The T-helper 17 recruitment factor CCL20 is a chemoattractant for lymphocytes and neutrophils, which may assist mucosal lymphoid tissues to attract lymphocytes and dendritic cells towards epithelial cells [8]. It is found on inflamed gut mucosa and its ligand CCR6 is found on T-helper 17 cells. Both CCL20 and CCR6 are upregulated in IBD [25,26]. Our results (previous [7] and current (Table 1)) suggest that T-helper 17 cell activity and migration to the site of colitis is suppressed by AA via *CCL20* gene downregulation at both time points (3 days post-AA and 28 days post-AA) [7,8], which makes CCL20 an attractive therapeutic target possibility.

The chemokine CCL5 attracts monocytes, memory T helper cells, and eosinophils to a specific site [17]. It induces histamine release from basophils and activates eosinophils [17]. At 28 days post-AA, *CCL5* was significantly upregulated (Table 1). Moreover, seven *CCL5*-associated gene sets were upregulated 28 days post-AA in contrast to one gene set downregulated (Table 3). This makes CCL5 an excellent potential therapeutic molecule to test on animal colitis models despite the possibility of pro-allergic physiology.

The chemokine CXCL11 is induced by gamma and beta interferons and is a chemoattractant for interleukin-activated T-cells [21]. Three days and 28 days post-AA *CXCL11* was significantly upregulated. On GSEA, six *CXCL11*-associated gene sets were upregulated in contrast to no gene sets downregulated. This makes the administration of exogenous CXCL11 a satisfactory therapeutic possibility to test on animal colitis models.

The chemokine CCL17 attracts and activates T lymphocytes [20]. Twenty-eight days post-AA, *CCL17* gene expression was significantly downregulated. Competitive inhibition of CCL17 by peptides is a new therapeutic approach to test on animal colitis models.

The chemokine CCL7 is a chemo-attractant for macrophages and a substrate of extracellular matrix metalloproteinase 2 [18] while CCL8 is a chemoattractant for monocytes, lymphocytes, basophils, and eosinophils [19]. The chemokine genes *CCL7* and *CCL8* showed high-fold-change expression increases in the distal colon three days post-AA when compared to three days post-SS (Table 1). However, these were not statistically significant. Therefore, GSEA analysis was done on *CCL7* and *CCL8* 28 days post-AA, but was unremarkable (Table 4). Additionally, *CCL7* and *CCL8* were chosen for further investigation for gene-expression changes by RT-PCR both three days post-AA and 28 days post-AA (Figure 1). RT-PCR expression of both *CCL7* and *CCL8* were significantly increased by AA three days after the second surgery (Figure 1). However, RT-PCR expression of *CCL7* alone was significantly decreased by AA 28 days after the second surgery (Figure 1). Therefore, CCL7 or CCL8 was not considered for follow-up studies as possible therapeutic targets.

All known chemokine receptor genes [23] were examined for gene expression levels in distal colons three days post-AA and 28 days post-AA. Interestingly and intuitively, but logically (chemokines induce upregulation of target and non-target chemokine receptors), there was no expression of any chemokine receptor gene three days post-AA. However, evidence of expression of most chemokine receptor genes occurred 28 days post-AA (Table 2). The chemokine receptor *CCR10* was the only chemokine receptor gene that displayed significant differential gene expression (upregulation) 28 days post-AA (Table 2). CCR10 is the receptor for CCL27, which attracts memory T lymphocytes [19]. However, using GSEA to dissect further, no *CCR10*-associated gene-sets were upregulated in contrast to one gene-set downregulated (Table 3). Therefore, CCR10 was not considered for follow-up studies as a possible therapeutic target.

To summarize, we have identified three new anti-colitic targets. The chemokines CCL5 and CXCL11 are direct therapeutic possibilities. The chemokine CCL17 is a potential target to be competitively inhibited.

## 4. Materials and Methods

### 4.1. Animal Experiments

Specific pathogen free Balb/c mice (Male, 5 weeks) were intraperitoneally anaesthetized with xylazine (5 mg/kg) and ketamine (100 mg/kg), which was followed by randomized allocation into two treatment groups, which included the appendicitis group or the sham surgery group [27]. Appendicitis was induced by constructing an appendiceal pouch from the caecal lymphoid patch and obstructing this by rubber band ligation using standardized negative aspiration. Sham surgery entailed a similar procedure, but, without continuous obstruction by band ligation of the caecal patch, the placement of a sterile rubber band in the abdominal cavity acted as a control for foreign body reaction. Seven days following the initial surgery, mice with appendicitis underwent an appendectomy (appendicitis and appendectomy, AA, group) while the control sham mice underwent a second sham surgery (sham and sham, SS, group). All mice were monitored daily. Three days after the second surgery, mice from both groups (SS group and AA group) were euthanized and the distal-most transmural colonic segments were harvested.

### 4.2. Processing of Colonic Specimens for RNA Extraction

Transmural colonic samples were cleaned promptly with normal saline and transferred to a TRIzol^®^ reagent (Invitrogen Australia Pty Limited, Mulgrave, Australia), snap-frozen in liquid nitrogen, and stored at −80 °C until microarray analysis was completed. Further extraction entailed chloroform and isopropanol treatment and centrifugation followed by washing the resultant pellet with 75% ethanol, drying, and re-constitution in nuclease-free H_2_O. Concentration and purity of RNA were determined by automated optical density evaluation (OD 260/OD 280 ≥ 1.8 and OD 260/OD 230 ≥ 1.8) using Nanodrop ND-1000 (Nanodrop Technologies, Wilmington, DE, USA). The degree of RNA degradation was analyzed by the Agilent electrophoresis bio-analyzer 2100 (Agilent Technologies Inc., Santa Clara, CA, USA) with the RNA integrity number (RIN) values consistently above 7.

### 4.3. Experimental Design of Microarray Study and the Affymetrix Array Process

All experiments were designed to be compliant with minimum information about a microarray experiment (MIAME) standards [28,29]. For Affymetrix array experiments, four individual test samples (for 3-day post-SS/AA time-point) or 3 individual test samples (for 28-day post-SS/AA time-point) were used per group (AA group versus SS group, one colonic sample per mouse) with each sample hybridized to an individual slide. The extracted RNA from each mouse specimen was taken individually through the microarray process. For Affymetrix arrays, 100 ng of RNA from each sample was labeled using the Whole Transcript Sense Target Labelling Assay as described (Affymetrix). Labeled cRNA samples were then hybridized to the Affymetrix Mouse Gene 1.0 ST Arrays (28,853 well-annotated genes) before being scanned. The Gene Expression Omnibus accession number for microarray data reported here is GSE23914 and the relevant link is http://www.ncbi.nlm.nih.gov/geo/query/acc.cgi?acc=GSE23914. All non-control probe sets from the eight arrays were imported into Partek (Version 6.4, Partek Inc., Chesterfield, MO 63005, USA) and then normalized using Robust Multi-Array Average [30]. The probability of each probe set being expressed was determined using the detected above background procedure and utilizing Affymetrix Power Tools (version 1.10.2). Probe sets were excluded if none of the samples were detected above the background level. To assess the degree of differential expression between AA and SS groups, a two-way Analysis of Varianceon treatment and batch was fitted to each probe set using Partek. To correct for multiple hypothesis testing, we used the q value/positive False Discovery Rate (FDR) [31].

### 4.4. Gene Set Enrichment Analysis and Enrichment of Chemokine and Chemokine Receptor Associated Gene Sets

We compared gene expression profiles from our garnered data to the c2_all collection of curated gene-sets (experimentally derived and from curated pathways) from the molecular signatures database (version 2.5) [22]. This approach termed GSEA, developed by Mootha [32], merges data from groups of gene sets previously described in literature to detect significant expression differences. A pre-ranked file was created containing the average difference between AA and SS for each probe set, which was sorted from most up-regulated in SS to most down-regulated. We used the na28 annotation csv file from www.affymetrix.com to determine the gene symbol for each probe set and collapsed probe sets to unique genes using the default, max_probe option, which resulted in 18,600 unique genes. GSEA (version 2.0, Broad Institute, Inc., MIT, Cambridge, Massachusetts, USA: and Regents of the UC, San Diego, California, USA) [22] was run in the pre-ranked mode using default parameters as elaborately described earlier [24]. Stringent statistical cut-offs (FDR values < 1% and *p* value < 0.001) determined chemokine associated gene-sets and chemokine receptor associated gene-sets, which were altered across distal colons of all AA mice when compared to all control SS mice.

### 4.5. Quantitative RT-PCR Expression of CCL7 and CCL8

We verified gene expression for the genes *CCL7* and *CCL8* via RT-PCR studies. Specific methodologies and the main RT-PCR study validation results have already been published [24].

### 4.6. Other Statistics Used

Group comparisons (other than the ones mentioned earlier) were analyzed using the Mann-Whitney U test with a GraphPad Prism (Graphpad software, San Diego, CA, USA). Data are expressed as a mean ± standard error of mean and the differences were considered to be significant if the *p* value < 0.05.

## 5. Conclusions

We have identified CCL5, CXCL11, and CCL17 as three new therapeutic targets to manipulate towards ameliorating or impeding colitic pathophysiology in animal models and potentially later in the mollification of human IBD. Both CCL5 and CXCL11 are satisfactory therapeutic molecule candidates to test initially on animal colitis models. Inhibition of CCL17 by peptides, antibodies, or small molecules is an exciting new therapeutic approach to test on animal colitis models.

## Figures and Tables

**Table 1:** Individual distal colonic gene expression described chemokine genes three days post-AA and 28 Days post-AA.

No.	Chemokine Gene	Other Names for Chemokine	Corresponding Chemokine Receptors	3-Day Post-AA		28-Day Post-AA	
				Fold-Change	*p*-Value	Fold-Change	*p*-Value
	**C Chemokines**						
1	*XCL1*	lymphoactin a, SCM-1a, ATAC	XCR1	-	-	1.03	0.709
2	*XCL2*	lymphoactin b, SCM-1b, ATAC	XCR1	-	-	-	-
	**CC Chemokines**						
1	*CCL1*	I-309	CCR8	0.95	0.344	0.99	0.870
2	*CCL2*	MCP-1, MCAF	CCR2	1.48	0.347	-	-
3	*CCL3*	MIP-1α, LD78α	CCR1, CCR5	1.19	0.125	-	-
4	*CCL4*	MIP-1β, LAG-1, ACT-2	CCR5	0.90	0.990	1.08	0.209
5	*CCL5*	RANTES	CCR1, CCR3, CCR5	0.91	0.537	1.22	0.048*
6	*CCL7*	MCP-3	CCR1, CCR2, CCR3	1.52^#^	0.150	0.85	0.164
7	*CCL8*	MCP-2	CCR3	1.87^#^	0.349	1.18	0.368
8	*CCL11*	eotaxin	CCR3	1.27	0.059	1.07	0.458
9	*CCL13*	MCP-4	CCR2, CCR3	-	-	0.95	0.721
10	*CCL14*	HCC-1	CCR1	-	-	-	-
11	*CCL15*	HCC-2, Lkn-1, MIP-1d, MIP-5	CCR1, CCR3	-	-	-	-
12	*CCL16*	HCC-4, LEC, LMC, LCC-1	CCR1	-	-	-	-
13	*CCL17*	TARC	CCR4	1.02	0.864	0.83	0.047*
14	*CCL18*	DC-CK1, PARC, AMAC-a, MIP-4	?	-	-	-	-
15	*CCL19*	MIP-3β, ELC, exodus-3	CCR7	0.98	0.667	0.95	0.554
16	*CCL20*	MIP-3α, LARC, exodus-1	CCR6	0.60	0.028*	0.63	0.023*
17	*CCL21*	6Ckine, SLC, exodus-2	CCR7	1.14	0.359	1.11	0.353
18	*CCL22*	MDC, STCP-1	CCR4	0.91	0.407	1.10	0.351
19	*CCL23*	MPIF-1, MIP-3, CKb-8	CCR1	-	-	-	-
20	*CCL24*	MPIF-2, eotaxin-2, CKb-6	CCR3	1.13	0.399	0.88	0.189
21	*CCL25*	TECK, MIP-4a	CCR9	0.90	0.285	0.90	0.566
22	*CCL26*	eotaxin-3	CCR3	0.91	0.153	0.96	0.496
23	*CCL27*	Eskine, CTACK, ILC	CCR10	1.04	0.497	1.03	0.710
	**CXC Chemokines**						
1	*CXCL1*	GROa, MGSA-a	CXCR1, CXCR2	1.03	0.680	0.93	0.292
2	*CXCL2*	GROb, MGSA-b, MIP-2a	CXCR2	1.08	0.460	1.01	0.913
3	*CXCL3*	GROg, MGSA-g, MIP-2b	CXCR2	0.98	0.786	1.07	0.463
4	*CXCL4*	PF4, oncostatin A	?	-	-	-	-
5	*CXCL5*	ENA-78	CXCR2	1.53	0.376	-	-
6	*CXCL6*	GCP-2	CXCR1, CXCR2	-	-	0.99	0.872
7	*CXCL7*	NAP-2, PPBP	CXCR2	-	-	-	-
8	*CXCL8*	IL-8, NAP-1, NAF, MDNCF	CXCR1, CXCR2	-	-	-	-
9	*CXCL9*	Mig	CXCR3	1.20	0.487	1.15	0.368
10	*CXCL10*	IP-10	CXCR3	1.82	0.324	1.50	0.100
11	*CXCL11*	I-TAC	CXCR3	1.17	0.044 *	1.16	0.043 *
12	*CXCL12*	SDF-1α/β	CXCR4	1.21	0.111	1.12	0.171
13	*CXCL13*	BLC, BCA-1	CXCR5	1.05	0.927	0.85	0.086
14	*CXCL14*	BRAK	?	1.13	0.215	0.83	0.085
	**CX3C Chemokines**						
1	*CX3CL1*	fractalkine	CX3CR1	1.00	0.995	0.89	0.161

All known chemokine genes [13] were examined for gene expressions levels in distal colons three days post-AA and 28 days post-AA. At three days post-AA, *CXCL11* was significantly upregulated and *CCL20* was significantly downregulated. At 28 days post-AA, *CCL5* and *CXCL11* were significantly upregulated and *CCL17* and *CCL20* were significantly downregulated. The SS group, sham and sham group, AA group, Appendicitis, and appendectomy group. The 3 days post-AA study used 4 AA mice versus 4 SS mice. The 28 days post-AA study involved 3 AA mice versus three SS mice. * *p* Value < 0.05. **^#^** High fold change. The chemokine list is based on descriptions from Zlotnick and Yoshie, 2000 [12].

**Table 2:** Individual distal colonic gene expression of 24 described chemokine receptor genes three days post-AA and 28 Days post-AA.

No.	Chemokine Receptor Gene	Other Names for Chemokine Receptor	3-Day Post-AA		28-Day Post-AA	
			Fold-Change	*p*-Value	Fold-Change	*p*-Value
	**Atypical Chemical Receptors (ACR)**					
1	*ACKR1*	CCBP1, GPD, Dfy, CD234	-	-	-	-
2	*ACKR2*	CCR10, D6, CCR9	-	-	-	-
3	*ACKR3*	RDC1, GPR159, CXCR7	-	-	0.93	0.231
4	*ACKR4*	CCR11, CCBP2, VSHK1, CCX-CKR, PPR1	-	-	1.01	0.867
5	*CCRL2*	HCR, CRAM-B, CKRX, CRAM-A, ACKR5	-	-	-	-
6	*PITPNM3*	NIR1, RDGBA3, ACKR6	-	-	-	-
	**C-C Motif Chemokine Receptors (CCR)**					
1	*CCR1*	CKR-1, MIP1aR, CD191	-	-	0.88	0.294
2	*CCR2*	CC-CKR-2, CKR2, MCP-1-R, CD192, FLJ78302	-	-	1.05	0.477
3	*CCR3*	CC-CKR-3, CKR3, CD193	-	-	1.11	0.143
4	*CCR4*	CC-CKR-4, CMKBR4, CKR4, k5-5, ChemR13, CD194	-	-	0.99	0.864
5	*CCR5*	CKR-5, CC-CKR-5, CKR5, CD195, IDDM22	-	-	1.02	0.737
6	*CCR6*	CKR-L3, GPR-CY4, CMKBR6, GPR29, DRY-6, DCR2, BN-1, CD196	-	-	0.85	0.145
7	*CCR7*	BLR2, CDw197, CD197	-	-	1.13	0.226
8	*CCR8*	CY6, TER1, CKR-L1, GPR-CY6, CDw198	-	-	0.93	0.506
9	*CCR9*	GPR-9-6, CDw199	-	-	1.06	0.685
10	*CCR10*		-	-	1.19	0.024 *
	**C-X-C Motif Chemokine Receptors (CXCR)**					
1	*CXCR1*	CKR-1, CDw128a, CD181	-	-	-	-
2	*CXCR2*	CMKAR2, CD182	-	-	-	-
3	*CXCR3*	CKR-L2, CMKAR3, IP10-R, MigR, CD183	-	-	0.93	0.419
4	*CXCR4*	LESTR, NPY3R, HM89, NPYY3R, D2S201E, fusin, HSY3RR, NPYR, CD184	-	-	1.13	0.373
5	*CXCR5*	MDR15, CD185	-	-	0.88	0.063
6	*CXCR6*	TYMSTR, STRL33, BONZO, CD186	-	-	1.14	0.101
	**C-X-3-C Motif Chemokine Receptors (CX3CR)**					
1	*CX3CR1*	CMKDR1, V28, CCRL1	-	-	1.13	0.218
	*X-C motif chemokine receptors (XCR)*					
2	*XCR1*	GPR5, CCXCR1	-	-	1.13	0.375

All known chemokine receptor genes were examined for gene expression levels in distal colons three days post-AA and 28 days post-AA. There was no gene expression of any chemokine receptor gene three days post-AA. There was gene expression of most chemokine receptor genes 28 days post-AA. *CCR10* was the only chemokine receptor gene that displayed differential gene expression (upregulation) 28 days post-AA. SS group, sham, and sham group. AA group, Appendicitis, and appendectomy group. The three-days post-AA study used 4 AA mice versus 4 SS mice. The 28 days post-AA study involved 3 AA mice versus 3 SS mice. * *p* Value < 0.05. The chemokine receptor list was based on descriptions from Nomiyama, Osada, and Yoshie, 2010 [23].

**Table 3:** Differentially regulated gene sets associated with differentially regulated individual chemokine and chemokine receptor genes in the distal colon 28 days post-AA.

Gene	Upregulated Gene-Sets in AA	No. of Enriched Genes	FDR *q*-val	Downregulated Gene-Sets in AA	No. of Enriched Genes	FDR *q*-val
**Differentially Regulated Chemokine Gene**						
***CCL5***	**UPREG 7 gene-sets**			**DOWNREG 1 gene-sets**		
	BOYLAN_MULTIPLE_MYELOMA_C_D_DN	253	0.036	MONNIER_POSTRADIATION_TUMOR_ESCAPE_UP	357	0.009
	BOYLAN_MULTIPLE_MYELOMA_PCA1_UP	100	0.016			
	LIANG_SILENCED_BY_METHYLATION_2	31	0.002			
	MAHADEVAN_RESPONSE_TO_MP470_UP	17	0.001			
	SABATES_COLORECTAL_ADENOMA_DN	260	0.016			
	SEITZ_NEOPLASTIC_TRANSFORMATION_BY_8P_DELETION_UP	68	0.000			
	WIELAND_UP_BY_HBV_INFECTION	86	0.002			
***CCL17***	**UPREG 0 gene-sets**			**DOWNREG 0 gene-sets**		
	-			-		
***CCL20***	**UPREG 1 gene-sets**			**DOWNREG 0 gene-sets**		
	LIANG_SILENCED_BY_METHYLATION_2	31	0.002	-		
***CXCL11***	**UPREG 6 gene-sets**			**DOWNREG 0 gene-sets**		
	MAHADEVAN_RESPONSE_TO_MP470_UP	17	0.001	-		
	RADAEVA_RESPONSE_TO_IFNA1_UP	30	0.009			
	SANA_RESPONSE_TO_IFNG_UP	56	0.000			
	SEITZ_NEOPLASTIC_TRANSFORMATION_BY_8P_DELETION_UP	68	0.000			
	UROSEVIC_RESPONSE_TO_IMIQUIMOD	15	0.023			
	WIELAND_UP_BY_HBV_INFECTION	86	0.002			
**DIFFERENTIALLY REGULATED CHEMOKINE RECEPTOR GENE**						
***CCR10***	**UPREG 0 gene-sets**			**DOWNREG 1 gene-sets**		
	-			SPIELMAN_LYMPHOBLAST_EUROPEAN_VS_ASIAN_UP	46	0.034

The gene set groups chosen for further evaluation had stringent cut-off values (FDR < 5% and *p* value < 0.001). Each entry had a *p* value of 0.000. Using these criteria, in our study, seven *CCL5*-associated gene sets were upregulated 28 days post-AA in contrast to one gene set downregulated. No *CCL17*-associated gene sets were upregulated or downregulated. One *CCL20*-associated gene set was upregulated in contrast to no gene-sets downregulated. Six *CXCL11*-associated gene sets were upregulated in contrast to no gene-sets downregulated. No *CCR10*-associated gene-sets were upregulated in contrast to one gene set downregulated. AA, appendicitis, and appendectomy group. UPREG “n” gene sets, upregulated number of gene sets pertaining to that gene. DOWNREG “n” gene sets, downregulated number of gene sets pertaining to that gene. No. of enriched genes in gene set, the number of genes in each gene set enriched by GSEA pertaining to AA mice in this study. FDR *q*-value, false discovery rate *q*-value statistic, GSEA, gene set enrichment analysis.

**Table 4:** Differentially regulated 28 days post-AA gene sets associated with chemokines CCL7 and CCL8 which showed high-fold-change gene expression in the distal colon three days post-AA.

Gene	Upregulated Gene-Sets in AA	No. of Enriched Genes	FDR *q*-val	Downregulated Gene-Sets in AA	No. of Enriched Genes	FDR *q*-val
***CCL7***	**UPREG 0 gene-sets**			**DOWNREG 1 gene-sets**		
	-			BERENJENO_TRANSFORMED_BY_RHOA_UP	495	0.006
***CCL8***	**UPREG 2 gene-sets**			**DOWNREG 0 gene-sets**		
	SABATES_COLORECTAL_ADENOMA_DN	260	0.016	-		
	UROSEVIC_RESPONSE_TO_IMIQUIMOD	15	0.023	-		

The gene set groups chosen for further evaluation had stringent cut-off values (FDR < 5% and *p* value < 0.001). Each entry had a *p* value of 0.000. Using these criteria, in our study, no *CCL7*-associated gene sets were upregulated 28 days post-AA in contrast to one gene set downregulated. Two *CCL8*-associated gene sets were upregulated in contrast to no gene sets downregulated. AA, appendicitis, and appendectomy group. UPREG “n” gene-sets, upregulated number of gene-sets pertaining to that gene. DOWNREG “n” gene-sets, downregulated number of gene-sets pertaining to that gene. No. of enriched genes in the gene set. the number of genes in each gene set enriched by GSEA pertaining to AA mice in this study. FDR *q*-value, false discovery rate *q*-value statistic, GSEA, gene set enrichment analysis.
